# Age-related changes in the immune system and challenges for the development of age-specific vaccines

**DOI:** 10.1080/07853890.2025.2477300

**Published:** 2025-03-20

**Authors:** T. Mark Doherty, Birgit Weinberger, Arnaud Didierlaurent, Paul-Henri Lambert

**Affiliations:** a GSK, Wavre, Belgium; b Universität Innsbruck, Institute for Biomedical Aging Research, Innsbruck, Austria; c Center of Vaccinology, University of Geneva, Geneva, Switzerland

**Keywords:** Vaccine, older adults, immunosenescence, inflammaging, adjuvant, Adjuvant system, AS01

## Abstract

**Background:**

A better understanding of how the immune system evolves with age and how vaccines work in older people has led to increasing focus on the development of vaccines aimed specifically at older age groups. We discuss strategies used to improve vaccine immunogenicity for older adults, focusing on licensed adjuvants.

**Findings:**

With age-related immune decline (immunosenescence), older adults face increased vulnerability to infections and severe complications. Immunosenescence affects T-cell and B-cell populations and innate immunity, leading to reduced chemotaxis, cytotoxicity, and altered cytokine production. This contributes to inflammaging—low-grade, chronic inflammation linked to aging. However, immune responses vary due to genetics and life-long exposures, making chronological age an imperfect indicator of immune health. Vaccination remains key to prevention, yet immune dysfunction complicates vaccine efficacy. Strategies to enhance responses in older adults include mRNA vaccines, high-antigen content vaccines, intradermal administration, and adjuvants. mRNA COVID-19 vaccines generated strong immune responses in older adults, though lower than in younger groups. High-antigen content influenza vaccines have shown superior efficacy compared to standard vaccination. Adjuvants offer a well-established approach to boosting vaccine responses by enhancing innate immunity.

**Conclusions:**

Of various strategies used to improve immunogenicity of vaccines for older adults, adjuvants have been the most consistently effective and practical. More recently, mRNA vaccines have also shown great promise.

## Background

Populations are aging in what seems to be an irreversible trend in many parts of the world. The United Nations projects that the number of people 65 years of age or older will increase from 761 million (1 in 10 people) in 2021 to 1.6 billion (1 in 6 people) in 2050 [1]. The percentage of the population at least 80 years of age is projected to rise even faster [1]. As populations age, ensuring that older adults remain healthy is of greater importance to both individuals and health care systems. Indeed, per capita health care costs in the United States (US) for those over 65 years of age are three to five times higher than for those under 65 years [2]. The concept of ‘healthy aging’ is the focus of the World Health Organization’s work on aging between 2015 and 2030, and is defined as ‘the process of developing and maintaining the functional ability that enables wellbeing in older age’ [3]. Preventive medicine needs to be a cornerstone of this approach.

Along with chronic diseases such as cancer and cardiovascular disease, infectious diseases are responsible for considerable morbidity and mortality in older adults and are still a common cause of death in individuals 60 years of age or older [4]. In countries with comprehensive infant and childhood vaccination programmes, the burden of vaccine-preventable diseases, including death, has declined in infants and young children but risen in older adults [5–7]. The rise in older adults likely results partly from the increasing proportion of older individuals in the population, but also from an increased incidence of infectious disease in this population. Data from Australia have shown that Disability Adjusted Life Years (DALYs)/100,000 population for vaccine-preventable disease fell in young children from 2005 to 2015, but rose among adults ≥50 years [6]. Older adults can be more vulnerable to some infections than younger adults and are more likely to experience severe illness, long-term complications, and hospitalisation [8]. For example, shingles (herpes zoster, HZ) is more prevalent in older people or in immunocompromised individuals, and older age is strongly associated with increased risk of zoster and of complications such as post-herpetic neuralgia [9,10]. Older people also experience more severe complications of respiratory infections such as influenza or coronavirus disease 2019 (COVID-19) [11–13]. The risks associated with respiratory syncytial virus (RSV) in older adults are less appreciated. Although the global burden of RSV is highest in children <5 years of age [14], RSV infection is common in older adults and mortality rates are considerably higher than in children [15,16]. Comorbidities, which are more common in older adults, increase the risk of some infectious diseases [17–20], and some infectious diseases have been shown to worsen underlying comorbidities [21,22].

The age-related decline in the immune system that leads to increased risk of infection and severe disease is also thought to be associated with an alteration of response to vaccination. Immunogenicity and effectiveness of some vaccines have been shown to be lower and to wane more rapidly in older than in younger people [23–26]. However, it is becoming increasingly recognised that factors other than age *per se* might be more important in determining the immune response to both infection and vaccination in older people (for example, genetics, overall health, frailty, and environmental factors) [27–29]. Recent research has shown that aging of the immune system is more complex than previously believed and, in some situations, might even provide some advantages over the immune systems of younger individuals [27–29]. A better understanding of how the immune system evolves with age and how vaccines work in older people has led to increasing focus on the development of vaccines aimed specifically at older age groups. This review aims to briefly outline how the immune system changes with age, how such changes vary between individuals, and the attributes of vaccines intended for older individuals, focusing on licensed vaccines.

## Aging and immunity

### Immunosenescence and inflammaging

Discussion of all the physiological changes leading to what is called immunosenescence is beyond the scope of this review. Briefly, immunosenescence is characterised by changes in both the number and relative balance of T-cell and B-cell populations. These changes lead to a fall in the proportion of naïve adaptive immune cells, the accumulation of memory T-cells, and a reduced ability to respond to new infections or vaccination [28,30]. Age-related changes in the innate immune system also occur, notably reduced chemotaxis, reduced cytotoxicity, and abnormal cytokine production [28,30]. These changes in immune cell distribution are thought to drive what is called “inflammaging” – a state of low-grade, chronic inflammation that develops with age [31,32].

Inflammaging is a risk factor for several age-related diseases, as well as for frailty and mortality [33,34]. It is associated with accumulation of cell debris as removal of senescent cells becomes less efficient, gut dysbiosis, increased risk of coagulation and fibrinolysis, the so-called senescence-associated secretory phenotype due to accumulation of senescent cells, and age-related innate immune system dysregulation [35]. Dysregulation of the innate immune system results in increased production of numerous cytokines and chemokines, including interleukin (IL)-6, IL1-ß, and tumour necrosis factor, leading to the establishment of the chronic low-level inflammation that gives inflammaging its name [35–38]. However, an age-related increase in anti-inflammatory mediators has also been demonstrated, indicating possible anti-inflammatory compensation and adaptation [32,39,40]. These findings suggest that the immune system adapts with age rather than simply declining [27].

Nonetheless, the immune dysfunction associated with immunosenescence and inflammaging might contribute to the pathogenesis of frailty, and in turn, people who experience frailty are more vulnerable to infection and serious complications. This relationship contributes to a vicious circle of degenerative disease [41,42].

### Immunobiography and immune fitness

**Figure 1.**
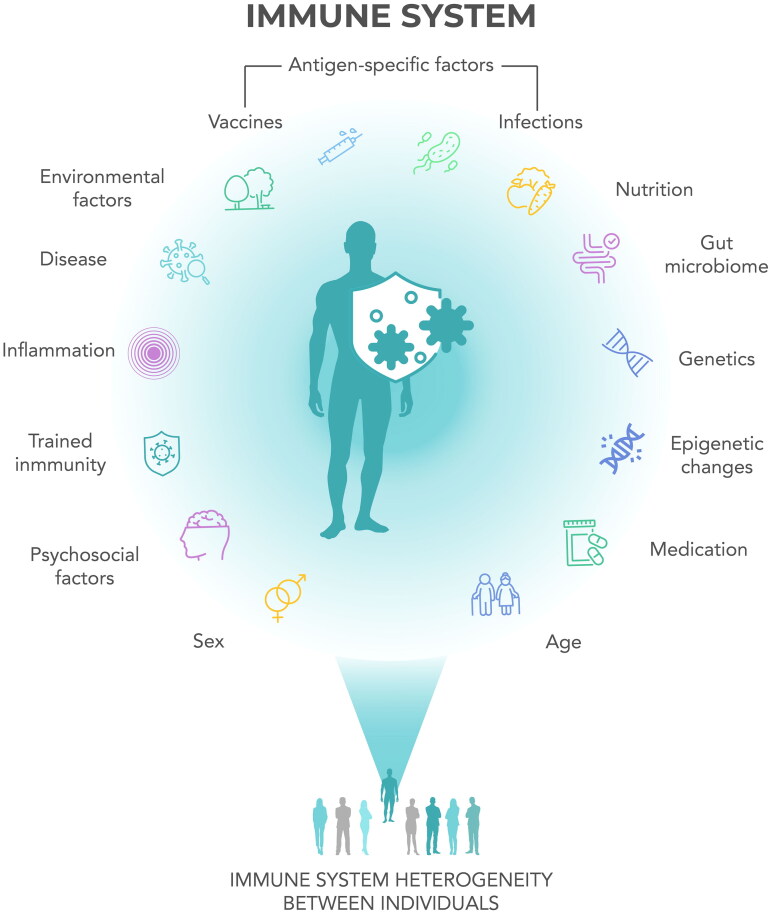
Individual immunobiography. The figure shows the factors that can influence the heterogeneity of the immune response, contributing to an individual’s immune biography

Although immunosenescence is a well-known concept, it is much less recognised that there is considerable heterogeneity in the immune response of older adults, influenced by both genetics and exposure to different external factors during the course of their lives [27,32]. The combination of such influences constitutes the immunobiography (or immune landscape) of each individual (Figure 1) [32]. External factors that influence the immunobiography include antigen-specific responses to infections and vaccinations and more general factors such as previous and current diseases, medications, diet, microbiome, and an almost unlimited range of environmental and lifestyle factors [32]. Epigenetic changes such as DNA methylation and histone acetylation that alter the accessibility of transcription factors and thereby influence gene expression provide a mechanism for longer-term induced changes to the immunobiography [43].

This variability in the immunobiography of individuals explains the heterogeneity in the immune response of older adults, meaning that chronological age is not necessarily correlated with biological age or immune health [32]. Thus, although aging is inevitable, a decline in immune fitness does not necessarily follow. An individual’s genetic make-up plays an important role in immune fitness, but external factors appear to be more influential [44]. A study of monozygotic twins showed that immunological parameters became more divergent in older age [44]. Data from the US Health and Retirement Study reported that T-cell measures of immunosenescence were higher in some racial and ethnic groups and among people with lower educational attainment – groups that have been shown to have higher risks for many infections [45]. An additional analysis found that psychosocial stress was associated with immunosenescence-related immune changes such as a decrease in naïve T-cells and an increase in terminally differentiated T-cells, independent of age, sex, or race [46].

A healthy lifestyle is therefore important in building and maintaining immune fitness which can slow down the process of immunosenescence and the development of frailty [28,29,42]. Vaccination can potentially contribute to immune fitness. Although its main aim is to protect against specific infections, vaccination can also have indirect beneficial effects. One such effect is its ability to help to maintain homeostasis by preventing infection-related long-term complications [28,29,42]. Examples of this effect include the reduction in risk for cardiovascular diseases, dementia, and ischaemic stroke associated with vaccination against influenza or HZ [47–52]. Another potential indirect benefit of vaccination is the induction of trained immunity whereby cells of the innate immune system are reprogrammed through the induction of epigenetic changes [28,29,53]. Well-described examples, where the effect is primarily seen in children, are the reductions in all-cause mortality with the measles or Bacillus Calmette-Guérin (BCG) vaccines – in these cases, the reduction in mortality is much greater than can be explained based on prevention of mortality due to measles or tuberculosis (TB) alone [54]. For measles, at least, this appears to be due to the detrimental effect of infection on immune function [55]. However, recent trials have indicated that the BCG vaccine does not offer consistent protection against COVID-19 in adults [56].

Knowledge of both the direct and indirect benefits of vaccination has contributed to the concept of life-course vaccination in which vaccination is seen as a life-long strategy to maintain better overall health and promote healthy aging [57]. At present, the relative contributions of the direct and indirect benefits of vaccination to overall health are unknown.

## Designing vaccines for older adults

The complex immune mechanisms needed to induce an efficient vaccine response often break down in older adults because of the age-related changes to the immune system described earlier. Thus, vaccines developed for children and younger adults are not always as effective in older adults and it may be necessary to design vaccines specifically tailored for older adults that take age-related changes into account [58]. Several strategies have been used to try to maximise the immune response to vaccination in older adults, including messenger ribonucleic acid (mRNA) vaccines, high-dose vaccines, intradermal vaccines, and adjuvanted vaccines.

### mRNA vaccines

The COVID-19 pandemic highlighted the vulnerability of older adults to respiratory infections and saw the widespread use of mRNA vaccines for the first time. Encouragingly, the two most widely used mRNA COVID-19 vaccines, which both used nucleoside-modified mRNA encoding an optimised variant of the spike protein found on the surface of the severe acute respiratory syndrome coronavirus 2 (SARS-CoV-2), generated strong T-cell and B-cell responses even in older adults; however, these responses were still lower than those documented in younger adults, particularly regarding T-cell responses, and frail individuals in particular showed reduced responsiveness [59–63]. Despite a lower immune response in older adults, real-world effectiveness was high, especially against more severe disease [59]. Although local and systemic reactions were common, particularly following the second dose, reactogenicity of the mRNA vaccines was considered acceptable [60,64].

Some studies have shown that the mRNA vaccines appeared to be more immunogenic in older adults than virally vectored or inactivated COVID-19 vaccines, something that may be attributable to improved antigen delivery and the intrinsic adjuvanticity of mRNA formulations, probably due to a combination of RNA and lipid nanoparticles [60,65]. In contrast, one study indicated that immunosenescence had a greater negative impact on adaptive immune responses induced by a mRNA vaccine compared with a vectored vaccine [62]. Immunity induced by mRNA vaccination against SARS-CoV-2 appeared to wane relatively rapidly [59]. The mechanism behind this waning is unknown, although it may be specific to the spike antigen rather than related to the mRNA vaccine platform itself. It is apparently not due to immune exhaustion or inability to produce memory responses, since immunity can be restored by booster vaccination [66,67]. It is worth noting in this context that even natural infection with SARS-CoV-2 does not appear to induce long-lasting immunity and that infection-induced immunity can also be boosted by mRNA vaccination [68]. Thus, while we can say that mRNA vaccines appear to be highly immunogenic in older adults, which is potentially promising for the development of adult vaccines, more work is required to establish whether mRNA vaccines can induce more durable responses with other antigens and whether formulation changes could maintain high immunogenicity while reducing reactogenicity of future candidate vaccines.

### High-dose strategies

High-dose vaccines are based on the premise that higher antigen content induces a more robust immune response than a standard antigen content. A potential drawback of the high-dose strategy is that the higher antigen demand might lead to higher prices and strain capacity for antigen production. A relevant example of this approach is the high-dose influenza vaccine, widely used in older adults, young children, and adults at risk of severe complications (e.g. pregnant women). This vaccine is currently recommended for universal vaccination in several countries [69,70]. Influenza vaccines are either trivalent or quadrivalent; trivalent vaccines contain two influenza A strains (A/H1N1 and A/H3N2) and one influenza B strain (B/Victoria) and quadrivalent vaccines contain an additional B strain (B/Yamagata). Several different formulations are available and the formulation used is tailored to the population. High-dose influenza vaccines are targeted at older populations, while younger adults and children usually receive standard-dose vaccines [69,71]. The high-dose formulations contain 45 μg or 60 µg haemagglutinin antigen for each virus strain, while the standard-dose formulations contain 15 µg per strain [71].

**Figure 2.**
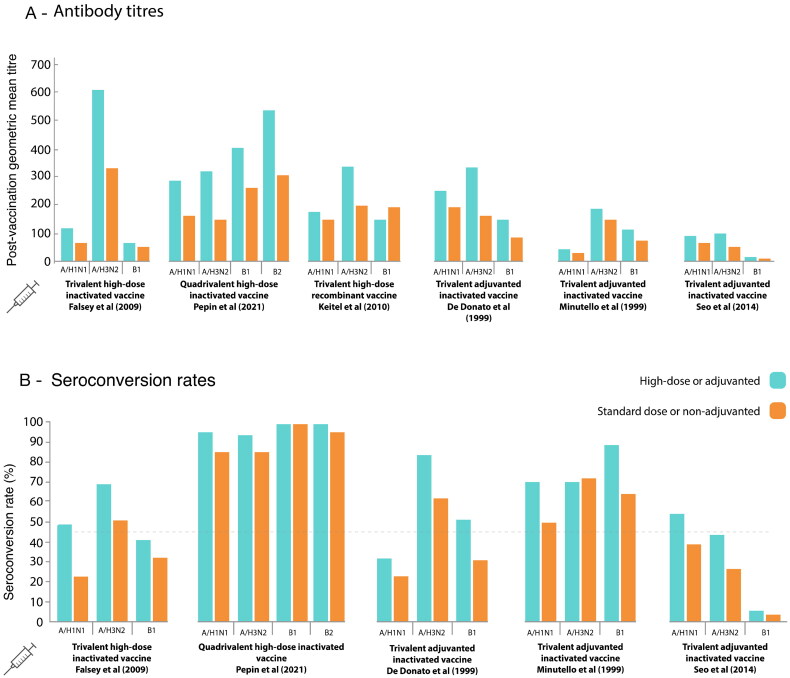
Antibody titres and seroconversion rates at 28 days following influenza vaccination in adults ≥65 years of age: comparison of standard vaccines with high-dose and adjuvanted vaccines. Falsey et al. (2009) [72], Pepin et al. (2021) [73], Keitel et al (2010) [74], De Donato et al (1999) [97], Minutello et al (1999) [98], Seo et al (2014) [99] The figure shows a comparison of antibody titres and seroconversion rates induced by standard vaccines versus high-dose and adjuvanted vaccines

Randomised trials in older adults have shown that high-dose influenza vaccines elicit higher antibody titres and higher seroconversion rates than standard-dose vaccines (Figure 2) [72–74]. In line with improved immunogenicity, high-dose vaccines have demonstrated superior clinical effectiveness in prevention of influenza disease compared with standard formulations [75–79]. A systematic review and meta-analysis showed that the effectiveness in prevention of influenza-like illness of the high-dose influenza vaccine relative to the standard-dose vaccine (i.e. the additional benefit of the high-dose vaccine) was 15.9% (95% confidence interval [CI]: 4.1, 26.3) [75]. The high-dose vaccine also provided superior protection against hospital admission and death [75].

The live attenuated varicella-zoster virus (VZV) vaccine is also used both in a low-dose formulation for children or adolescents for the prevention of primary varicella infection (chickenpox) and in a high-dose formulation for older adults to prevent reactivation of VZV and subsequent HZ (shingles). However, since the presentation and immunopathology of chickenpox and HZ are quite different, comparisons are difficult to draw. The use of the high-dose live attenuated VZV vaccine is discussed below in more detail in the section of HZ vaccines.

### Intradermal vaccination

Intradermal vaccination exploits the abundance of antigen-presenting cells in the skin to increase antigen presentation and thus enhance downstream immune responses [80]. A systematic review and meta-analysis found that intradermal influenza vaccine at a dose of 15 µg haemagglutinin offered higher seroconversion rates and higher antibody titres against influenza B than an intramuscular vaccine at an equivalent dose in adults ≥60 years of age [81]. However, the immunogenicity of the intradermal and intramuscular formulations was similar for both influenza A strains [81].

Although intradermal vaccination may hold promise for inducing more robust immune responses, there are challenges in delivering vaccines by this route in older people, including skin fragility and a lower number of antigen-presenting cells in older skin [41]. Studies of influenza vaccination in older adults suggested that there are insufficient data to support a conclusion of improved immunogenicity of intradermal vaccination in persons aged 65 years or older and this practice is no longer recommended in countries such as the US [82]. However, a small study in Japan, comparing intramuscular and intradermal vaccination with live attenuated VZV vaccine did find significantly increased VZV-specific T-cell memory in the intradermal injection group, and this was associated with increased Th1, Th2 and Th17 cytokine expression [83]. Some studies in the meta-analysis mentioned above found enhanced immunogenicity compared with intramuscular injection for influenza vaccination carried out in cohorts predominantly comprised of younger, healthy adults [81]. It should also be noted that studies of intradermal hepatitis B vaccination (primarily in infants or younger, healthy adults) did not find increased immunogenicity compared with intramuscular injection – instead, there was a tendency to lower responsiveness [82,84]. This highlights the difficulty of extrapolating across different vaccine formulations and different populations.

### Adjuvant technology

Adjuvants are substances included in vaccines to boost the magnitude, durability and quality of the immune response *via* various mechanisms [41]. They have been used in vaccines for decades and are a well-characterised and consistent way of improving the immune response to vaccines *via* activation of the innate immune response. Adjuvants included in currently licensed vaccines include alum, MF-59, cytosine-phosphorothioate-guanine-oligodeoxynucleotide (CpG), Toll-like receptor (TLR)7 ligand, Matrix-M, and the Adjuvant System (AS) family of adjuvants [85–91].

#### MF-59

MF-59 is an oil-in-water emulsion that contains squalene [85]. It is known to induce pro-inflammatory cytokines and chemokines that activate dendritic cells and to enhance antigen uptake by antigen-presenting cells and their transport to draining lymph nodes [92]. It also induces the release of adenosine triphosphate in muscle, which in turn enhances antibody responses to vaccine antigens and drives germinal centre B-cell differentiation [93–95]. When used in influenza vaccines, it has been shown to induce cross-reactive immune responses against non-vaccine strains, as well as against the stalk component of the haemagglutinin protein [96].

Randomised, controlled trials in older adults have shown that influenza vaccines adjuvanted with MF-59 induce a more robust immune response than non-adjuvanted influenza vaccines (Figure 2) [97–99]. In a meta-analysis of adjuvanted versus non-adjuvanted vaccines, older adults (>64 years) receiving influenza vaccines adjuvanted with MF-59 had a 26% higher likelihood of achieving seroconversion against the A/H1N1 influenza virus strain than those receiving non-adjuvanted vaccines (relative risk: 1.26, 95% CI: 1.10, 1.44) [100].

The clinical benefit of MF-59-adjuvanted influenza vaccine over non-adjuvanted vaccine has also been demonstrated. A systematic review and meta-analysis of the effectiveness of MF59-adjuvanted versus non-adjuvanted influenza vaccines in adults ≥65 years demonstrated that the absolute efficacy of the adjuvanted vaccine was 40.7% for reduction of outpatient office visits and 58.5% for reduction of hospitalisation [101]. The effectiveness of the adjuvanted vaccine relative to the non-adjuvanted vaccine was 13.9% (95% CI: 4.2, 23.5) for prevention of any influenza-related medical encounters (outpatient office visits, hospitalisation, emergency room visits) [101].

Analyses directly comparing adjuvanted versus high-dose influenza vaccines have indicated overall similar immunogenicity and clinical efficacy [101–106]. A systematic review and meta-analysis of the effectiveness of MF-59-adjuvanted influenza vaccine compared with non-adjuvanted high-dose influenza vaccine in adults ≥65 years of age found no consistent benefit of one vaccine over the other [106]. For example, the adjuvanted vaccine was more effective than the high-dose vaccine in reducing all influenza-related medical encounters (relative effectiveness 9.7% [95% CI: 5.0, 14.2]) but was less effective than the high-dose vaccine in reducing hospitalisation for any respiratory condition (relative effectiveness −13.9% [95% CI: −25.4, −3.4]) [106]. Overall, both vaccines appeared to have similar effectiveness against influenza in older adults [106].

#### Adjuvant system AS01

##### Composition and mode of action

In natural infection, multiple pathways of the innate immune system are activated simultaneously. Adjuvant Systems combine several immunostimulants to activate different parts of the innate immune system, resulting in complementary and even synergistic immunostimulatory activities [88]. One member of the Adjuvant System family, AS01, is used in several vaccines for older adults. It is a liposome-based adjuvant comprising 3-*O*-desacyl-4′-monophosphoryl lipid A (MPL), a Toll-like receptor 4 (TLR4) ligand and QS-21, a saponin extracted from the bark of the *Quillaja saponaria* Molina tree [88,91]. MPL signals through TLR4, promoting two different signalling pathways: MyD88 (myeloid differentiation primary response 88) and TRIF (TIR-domain-containing adapter-inducing interferon [IFN]-β) [91,107]. MyD88 activates nuclear factor-kappa B (NF-κB) which induces transcription of NF-κB-dependent genes and cytokines, while TRIF promotes production of IFN-γ [91,107]. TLR4 also plays a role in maturation of dendritic cells and monocytes [91,107,108]. The entry of QS-21 into cells is cholesterol-dependent and leads to Syk-pathway activation and destabilisation of the lysosomal membrane and the release of inflammatory stress molecules such as high mobility group box 1 protein and cathepsin B involved in the NLRP3 inflammasome activation. Both subcapsular macrophages, as target cells of QS-21 and Myd-88-dependent signalling, are required for the QS-21 mediated induction of humoral and cellular responses [91,109,110].

**Figure 3.**
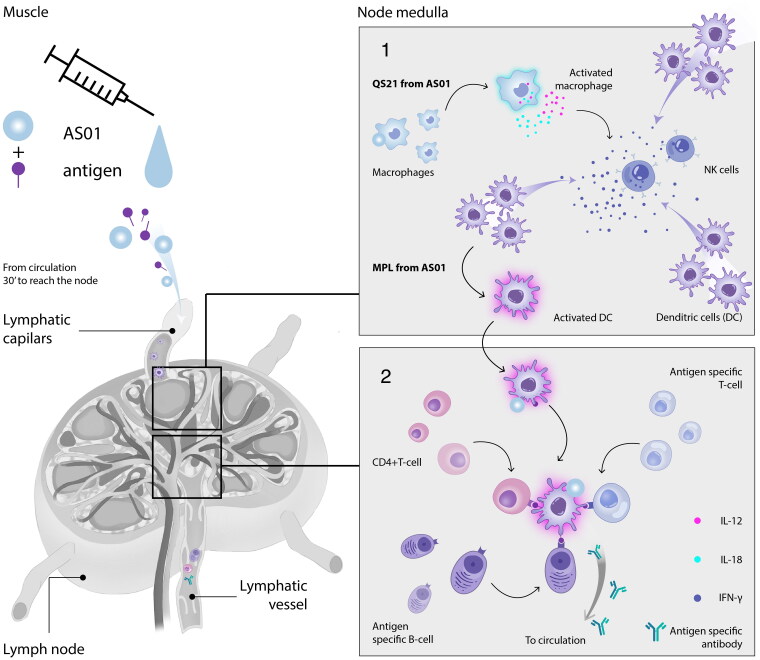
Mechanism of action of AS01. Amended from Cunningham et al. 2021 [111] The figure illustrates the mechanism of action of AS01, showing how QS21 stimulates macrophages to release IL-12 and IL-18, which in turn stimulate NK cells to release IFN. IFN attract dendritic cells which are stimulated by MPL. The dendritic cells then present antigen to both B-cells and T-cells. AS, Adjuvant System; DC, dendritic cell; IFN, interferon; IL, interleukin; MPL, 3-*O*-desacyl-4′-monophosphoryl lipid A; NK, natural killer

There is a synergistic effect of combining MPL and QS21 in AS01 [88,91,111]. In mouse models, AS01 reaches the lymph node draining the injection site within 30 min; there, QS21 stimulates macrophages to release IL-18, and together with IL-12 stimulate natural killer cells and CD8 T-cells to release IFN-γ (Figure 3) [88,91,111]. This further activates dendritic cells in the lymph node that were recruited from the muscle and blood. These fully activated dendritic cells then present the vaccine antigen to T-cells [91,111]. The combination of MPL and QS-21 therefore leads to a synergistic activation of innate immunity leading to an IFN-γ-dependent induction of polyfunctional T-cells. Those T-cells further support an enhanced antibody response through help to antigen-specific B-cells. In humans, a core gene signature has been identified for several AS01-adjuvanted vaccines, characterised by the upregulation of IFN-stimulated genes, monocyte activation and negative regulation of genes related to natural killer cells [112–114]. It is believed that the core gene signature might be common to AS01-adjuvanted vaccines in general [115]. Recently, an AS01-adjuvanted hepatitis B virus vaccine was shown to elicit trained immunity in monocytes, as shown by adjuvanted influenza vaccines [53].

AS01 is used in an HZ vaccine, an RSV vaccine, a candidate hepatitis B vaccine, a candidate TB vaccine, and a malaria vaccine. All are used or intended for use in older adults except the malaria vaccine which is used exclusively in children.

##### AS01-adjuvanted HZ vaccine

Varicella (chickenpox) and HZ (shingles) are two different diseases caused by the same virus (VZV) [116,117]. During primary infection, the virus enters sensory nerves and becomes latent in the nervous system [116,117]. Primary infection causes varicella, usually in children, whereas reactivation of the latent virus causes HZ, usually in older adults [116,117]. The incidence of HZ increases considerably after 50 years of age [118–120].

The varicella vaccine used in children is a live attenuated vaccine consisting of a minimum of 1350 plaque forming units (PFU) of the Oka VZV strain [121]. The Zoster Vaccine Live (ZVL) used in adults to prevent HZ uses a high-dose strategy and consists of a minimum of 19,400 PFU of the same virus strain, 14 times the amount in the varicella vaccine [121]. It was first licensed in 2006 in the US. Phase 3 licensure trials of ZVL demonstrated vaccine efficacy against HZ of 63.9% in adults aged 60–69 years, but lower efficacy of 37.6% in those aged ≥70 years (overall efficacy was 51.3% [95% CI: 44.2, 57.6]) [122]. Efficacy of the vaccine over the long term was not sustained, with overall vaccine efficacy declining to 21.1% over follow-up from 7 years through to 11 years post-vaccination [123]. Real-world studies also demonstrated rapidly declining vaccine effectiveness [124,125].

An AS01-adjuvanted recombinant zoster vaccine (RZV) containing a recombinant VZV glycoprotein E was first approved in 2017 in the US [126]. Vaccine formulation development studies compared adjuvanted with non-adjuvanted formulations and found substantially higher immunogenicity with the AS01-adjuvanted formulation, particularly in older age groups [127]. Compared with a non-adjuvanted formulation, the adjuvanted vaccine induced 3.4-fold, 4.2-fold, and 10.6-fold higher antigen-specific T-cell responses in adults aged 50–59, 60–69, and ≥70 years, respectively [127]. The same pattern was seen for humoral immunity, with 2.7-fold, 5.1-fold, and 7.5-fold higher antibody responses seen in the same age groups versus non-adjuvanted vaccine [127]. Pivotal phase 3 trials demonstrated RZV vaccine efficacy against HZ of 91.3% in adults ≥50 years, 91.3% in adults 70–79 years and 91.4% in adults ≥80 years [128]. In the overall population (≥50 years), vaccine efficacy against HZ was reported as 97.6% at year 1 and 87.9% at year 4 [128]. Vaccine efficacy against post-herpetic neuralgia was 91.2% in adults ≥50 years and 88.8% in those ≥70 years [128]. An interim analysis of a long-term follow-up study reported overall vaccine efficacy of 81.6% up to almost 10 years post-vaccination [129]. Real-world effectiveness of RZV has also been demonstrated [130].

RZV is the first vaccine demonstrated to induce a robust cellular response in older adults. RZV is either the only HZ vaccine recommended or is the preferred HZ vaccine in the routine immunisation programme of several countries, including the US, Austria, Germany, the Netherlands, Spain, the United Kingdom, Canada, and Australia [131–134].

##### AS01-adjuvanted RSV vaccine

Clinical efficacy and safety of the AS01-adjuvanted RSV vaccine have been demonstrated in older adults [135]. Efficacy of one vaccine dose against RSV-related lower respiratory tract disease was 82.6% overall and 94.1% against severe disease in the first year post-vaccination [135]; over two seasons, one dose followed by a booster dose a year later resulted in vaccine efficacy of 67.1% and 78.8%, respectively, against overall and severe RSV-related lower respiratory tract disease [136]. The vaccine was licensed in 2023 in the US, Canada, Japan, the United Kingdom, and the European Union.

A phase 1/2 study evaluated immunogenicity of a non-adjuvanted RSV vaccine administered to young adults and older adults (60–80 years) and the AS01-adjuvanted RSV vaccine administered only to older adults [137]. Both adjuvanted and non-adjuvanted vaccines elicited a robust immune response; polyfunctional CD4+ cells were slightly higher with the adjuvanted than with the non-adjuvanted vaccine [137]. A further study evaluated neutralising antibody responses against a range of RSV strains, including contemporary and antigenically distant strains, and demonstrated that the AS01-adjuvanted RSV vaccine boosted broader neutralising antibody responses in older adults compared with a non-adjuvanted formulation [138]. The breadth of the immune response suggested that the AS01-adjuvanted vaccine might be able to protect against a range of RSV strains, including emergent strains [138].

##### Other AS01-adjuvanted vaccines

Several other AS01-adjuvanted vaccines are available, including vaccines against TB, HIV, and hepatitis B. Although these vaccines are not specifically aimed at older adults, it is interesting to see how characteristics of the immune response could be related to the effectiveness of AS01 in older people.

The AS01-adjuvanted TB vaccine has been shown to be immunogenic and offer protection against progression to pulmonary TB disease [139]. An AS01-adjuvanted HIV nanoparticle vaccine candidate is also in early development and has been shown to induce CD4+ helper T-cell responses [140].

An exploratory study designed to compare the immunogenicity of AS01 and other Adjuvant Systems (i.e. AS03 [oil and water emulsion] [89] or AS04 [MPL adsorbed on aluminium salt] [90]) combined with hepatitis B antigen showed that the AS01-adjuvanted formulation elicited the highest number of hepatitis B-specific CD4+ T-cells [141]. A study of the core gene signature found that it was related to the extent of hepatitis B-specific antibody response and was higher in individuals who received an AS01-adjuvanted formulation than an AS03 or AS04 formulation [112]. Importantly, the AS01-adjuvanted formulation induced the core signature in most vaccine recipients, suggesting that AS01 is able to mobilise innate immunity more efficiently than other adjuvants, which may explain its ability to consistently induce robust cellular responses, irrespective of the vaccine antigen [112].

##### Immune interference and trained immunity

As the use of vaccines in older adults increases, there is a theoretical possibility that successive administration of different vaccines containing the same adjuvant could lead to immune interference, whereby the immunogenicity of the second vaccine is decreased. Since AS01 acts mainly locally at the injection site, potential interference is unlikely if the vaccines are administered in opposite arms, unless a systemic effect of the vaccine (as measured in blood signature post-vaccination) is sufficient to induce trained immunity and a differential response to a subsequent vaccination. The possibility was investigated in a study evaluating co-administration of the AS01-adjuvanted RZV vaccine with another AS01-adjuvanted vaccine (in this case a candidate vaccine against chronic obstructive pulmonary disease, which is now discontinued). The study provided evidence against the immune interference hypothesis, showing similar immune responses regardless of whether the vaccines were administered alone or together at several month intervals [142]. A retrospective, observational study reported a reduced risk of COVID-19 disease in older adults who had received RZV, suggesting that AS01 might enhance the immune response to subsequent antigens by mechanisms not yet well defined but which may include trained immunity effects [143]. The impact of RZV on subsequent disease (including COVID-19) is being explored in nursing home cohort (NCT04523246). A study of monocytes isolated from participants in a clinical trial of AS01-adjuvanted hepatitis B vaccine showed that the vaccine increased the number of monocytes and induced epigenetic alterations that resulted in functional changes including an improved response to IFN [53]. The clinical relevance of this effect of an AS01-adjuvanted vaccine on monocyte responsiveness to IFN remains to be determined.

##### Safety and reactogenicity

The safety profile of existing adjuvanted vaccines is well established. Typically, adjuvanted vaccines are more reactogenic than non-adjuvanted vaccines (other than mRNA vaccines), resulting in a higher incidence of reactions such as pain and swelling at the injection site, likely as a result of the greater innate immune response induced by the adjuvant. A meta-analysis of vaccines containing the Adjuvant System family and MF59 reported that those reactions are transient and mild to moderate in nature and, importantly, there is no association with serious adverse events, long-term diseases or disabilities or deaths [144]. There is some evidence of dosing effects; for example, the incidence of marked reactogenicity (grade 3) appears to be lower with AS01-adjuvanted vaccines containing a lower dose of AS01 (25 μg) compared with those containing a higher dose (50 μg) [128,135].

## Conclusions

The global increase in the number of older adults highlights the importance of healthy aging, in which vaccination will play a key role. The design of appropriate vaccines for older adults is vital to overcome poor immunogenicity associated with age-related adaptation of the immune system function, as formulations that are effective in children and young adults cannot be assumed to be suitable for older adults. Of various strategies used to improve immunogenicity of vaccines for older adults, adjuvants have been the most consistently effective in older adults and the most generally practical for vaccine formulation; furthermore, there is an abundance of evidence of their safety and immunogenicity. More recently, mRNA vaccines have shown great promise in older adults. While head-to-head comparisons of adjuvanted vaccines versus mRNA vaccines are needed to determine the benefits of each approach, both are likely to be beneficial, and perhaps complementary, for older adults. Areas for future research include development of predictive animal models for testing vaccine efficacy in older people, development of biomarkers to stratify older adults based on their immune fitness to allow tailoring of vaccine strategies and optimisation of dose and administration schedules for different populations, reduction of reactogenicity, comparison of different vaccine platforms, and a better understanding of the interaction between individual adjuvants and antigens and between pre-existing immunity and the immune response elicited by the adjuvant.

## Data Availability

Data sharing is not applicable to this article as no new data were created or analyzed in this study.

## Trademark

AS01 and AS03 are trademarks owned by or licensed to GSK.
